# Identifying candidate ncRNAs that direct changes in chromatin structure

**DOI:** 10.1186/1756-8935-6-S1-P69

**Published:** 2013-03-18

**Authors:** Mridula K Ray, Yanqun Wang, Mark Borowsky, Ruslan Sadreyev, Robert E Kingston

**Affiliations:** 1 Department of Molecular Biology, Massachusetts General Hospital, Boston, MA 02114 USA & The Department of Genetics, Harvard Medical School, Boston, MA 02115, USA

## Background

Long non-coding RNAs (ncRNAs) are increasingly recognized as important regulators of genomic processes and cell specification. Many IncRNAs are hypothesized to localize to and regulate chromatin, functionally impacting epigenetic state through interactions with chromatin-modifying proteins’. Numerous IncRNAs have been reported to play a role in the activity or recruitment of the epigenetic factors the Polycomb group (PcG) proteins to genomic sites^2,3^. However, identification and functional validation of chromatin-interacting RNAs are technically challenging with respect to distinguishing RNAs that bind chromatin modifying machinery from artifacts.

## Materials and methods

We developed a method to enrich for bonafide ncRNA-PcG interactions on the DNA. Chromatin, the substrate of the PcG proteins, was purified away from the cytoplasm and soluble fraction of the nucleus in crosslinked HeLa cells. The PcG component Bmi1, and a stable FLAG-Bmi1 were then specifically immunoprecipitated from the chromatin extract. Bmi1-associated ncRNAs were biochemically isolated and further studied with the aim of validating and understanding the roles of individual IncRNAs in PcG biology. Cross-validated Bmi1-interacting candidates were compared with immunoprecipitations against replicate Bmi1 samples, additional PcG components, and other chromatin protein controls (CTCF, H3K27me3, H3K4me3).

## Results

We have identified strong candidates at known IncRNA sites that are expressed from loci with distinct chromatin signatures. These noncoding RNAs are specifically and reproducibly enriched across replicates. For comparison, no mRNA exons are enriched in both FLAG-Bmi1 and Bmi1 pulldowns. Since mRNA exons are usually enriched in canonical RNA immunoprecipitations, but are considered a major source of noise, this protocol effectively eliminates a large number of false positives. Validation of individual candidates is underway, including siRNA screening for candidates which affect Bmi1 localization (ChIP) or expression of Bmi1 targets (RNA-seq).

## Conclusions

This technology offers an advantage over current RNA immunoprecipitation techniques by enriching the input for ncRNA-chromatin interactions, cross-validating results and employing stringent wash conditions and controls. Application of this technology to a variety of chromatin proteins yields distinct spectra of candidate IncRNA-chromatin interactions, and will enable future identification of IncRNAs that coordinate with proteins to direct chromatin state.
